# Development and consensus of entrustable professional activities for final-year medical students in anaesthesiology

**DOI:** 10.1186/s12871-022-01668-8

**Published:** 2022-04-29

**Authors:** Andreas Weissenbacher, Robert Bolz, Sebastian N. Stehr, Gunther Hempel

**Affiliations:** Department of Anaesthesiology and Intensive Care, University of Leipzig Medical Centre, Liebigstrasse 20, 04103 Leipzig, Germany

**Keywords:** Undergraduate, Assessment, Competency-based medical education, Entrustable professional activities, Anaesthesiology

## Abstract

**Background:**

The transfer of classic concepts of competency-based medical education into clinical practice has been proven to be difficult in the past, being described as partially fragmented, misleading and inadequate. At the beginning of training, novice doctors commonly feel overwhelmed, overloaded and exposed to extreme time pressure. The discrepancy between expected and actual clinical competence of doctors at the start of their speciality training jeopardizes patient safety. The framework of Entrustable Professional Activities (EPAs) is a promising instrument to effectively integrate competency-based training into clinical practice and may help to close this gap and consequently to improve patient safety.

**Methods:**

For anaesthesiology, we developed 5 EPAs for final-year medical students. The EPAs comprised the following seven categories: 1. Title, 2. Specifications, 3. Limitations, 4. Competency domains, 5. Knowledge, abilities and skills, professional attitudes, 6. Assessment and 7. Entrustment. Based on a modified, online-based Delphi study, we further developed and refined these EPAs. Education experts were recruited from the alumni network of the *Master of Medical Education* (MME) degree course from the University of Heidelberg, Germany.

**Results:**

28 data sets were evaluated in three Delphi rounds. 82% of study participants had previous experience with EPAs. Qualitative and quantitative data formed the basis during the iterative process and resulted in complete descriptions of 5 EPAs for final-year medical students in anaesthesiology.

**Conclusions:**

Our study including the associated description of 5 EPAs represent a further step and starting point for EPA-based curricula in medical training in Germany linking undergraduate training, to residency training and continuous medical education.

**Supplementary Information:**

The online version contains supplementary material available at 10.1186/s12871-022-01668-8.

## Background

When starting clinical training, young doctors often do not fulfil the expectations of senior medical staff [1–3]. Novice doctors frequently feel professionally overwhelmed, overloaded with work and time pressure [4]. Discrepancies between expected clinical competencies and those that actually exist are a threat to patient safety [5]. As a result, competency-based medical education (CBME) has become increasingly important, focusing on outcomes rather than overall training time [6]. However, the focus on sole imparting and examination of competencies is being discussed more and more critically, especially if performed as training on the job. A number of authors therefore describe the approach as inadequate, fragmented and misguided [7–11].

Ten Cate and colleagues introduced the concept of Entrustable Professional Activities (EPAs) as an alternative to the classic competency-based training in order not only to improve training per se, but also to make a significant contribution to patient safety [12–14]. EPAs are an attempt to close the gap between individual competencies and sometimes complex clinical tasks [15]. They describe typical everyday clinical activities that include knowledge, skills and abilities as well as professional attitudes. These activities are then gradually entrusted to the trainee [16]. They range from simple tasks to complex sequences of activities on a professional level [17]. Several smaller EPAs, so-called nested EPAs, can be combined into a superordinate EPA to create continuity between undergraduate, graduate and speciality training [17]. An EPA should generally comprise seven categories: title, specification, limitation, competency domains, knowledge, abilities, skills and attitudes, assessment and entrustment level [17–19]. Competency domains are derived e.g. from the *CanMEDS Physician Competency Framework* [20]. The EPA framework allows supervisors to make evidence-based entrustment decisions, e.g. based on workplace-based assessments. It is important to systematically assess the ability of a learner to perform an activity competently and safely. Workplace-based exams are validated tools to effectively influence the behaviour of trainees through structured feedback [21, 22]. Due to direct connection to professional practice and the description of typical everyday activities, this concept is also easier to convey to students, supervising doctors and program directors [23].

EPAs can be entrusted to trainees at different supervision levels. These levels of supervision range from passive participation “only allowed to observe” to the decision to supervise others in the execution of these EPAs [24]. The following levels of supervision were postulated for student teaching [16, 25–28].

1. “Only allowed to observe”: The student observes the EPA directly but does not apply it to the patient.
2. "Direct supervision": The student performs the EPA on the patient under direct medical supervision (supervisor in the room).
  a EPA is carried out as a co-activity with the supervisor.
  b EPA is carried out independently under the supervision of the supervisor.
3. “Indirect supervision”: The student conducts the EPA under indirect medical supervision (supervisor not in the room).

Together with the EPA description, the levels of supervision can be used to operationalize competence-based training in a meaningful way [23].

In everyday clinical practice ad-hoc decisions are constantly made in the sense of entrusting a certain activity for a specific clinical situation (*ad-hoc decision making*) [5]. With *summative decision making*, ten Cate and colleagues describe that several trainers decide at different points in time regarding the entrustment of a certain EPA to a trainee. Based on this continuous supervision and evaluation of performance, a decision is made regarding the level of supervision at which the trainee is allowed to look after patients independently. This decision is EPA specific and are based on training interviews, structured observations and workplace-based assessments [5].

The EPA concept is increasingly being adapted, applied and evaluated in undergraduate and postgraduate training [27, 29–33]. There are strategies for developing core EPAs for a complete medical curriculum or specialist training as well as nested EPAs for individual areas or subspecialties. However, there are no data yet to show that the introduction of EPAs or an EPA-based curriculum can increase student competence or have a positive impact on patient care. Furthermore, Marty et al. were able to show that there is a large difference between the curricular expectations and the competence perceived by the students themselves in EPAs [34]. In the Netherlands, Wisman-Zarter and colleagues assembled EPA titles in a Delphi study for postgraduate speciality training in anaesthesiology [35]. EPAs were also introduced as a promising concept for speciality training in anaesthesiology for Germany [36]. To this date, complete EPAs have not been developed for medical students in the field of anaesthesiology. Therefore, the aim of this study is to identify, specify and assemble relevant EPAs for undergraduate medical studies. These nested EPAs are optional for all students doing a rotation in the field of anaesthesiology in their final year and they should also offer the possibility to be implemented in undergraduate-level training at universities as well.

## Methods

### Initial EPA definition

The first step in building an EPA-based curriculum is to identify the key EPAs of a profession [16]. An author group at the Department of Anaesthesiology and Intensive Care at the University of Leipzig Medical Centre initially identified and conceptualized five core EPAs for final-year medical students through a structured literature research. Additionally, members of the department’s teaching conference, stakeholders and representatives of the department were involved in this process. These core activities were to be performed independently under supervision of senior medical staff and comprise seven categories.

### Process definition and Recruitment of participants

Holzhausen and colleagues postulated a primarily online-based Delphi consensus procedure with three rounds as a pragmatic instrument for developing and consenting EPAs [23]. This is an iterative interaction between the authors and a group of medical education experts. We therefore carried out a modified, solely online-based Delphi study with education experts. These experts were recruited from the *Master of Medical Education (MME)* alumni network at Heidelberg University, Germany and were also affiliated with the field of anaesthesiology. 49 contact details could be extracted from the database of the alumni network. Firstly, all contact details were checked for validity. Five were subsequently adjusted due to workplace change. Invitation to the study, including detailed study information, was sent via email. The online-based approach offered the advantage that participants could evaluate the EPAs according to their own time frame. Set time slots would certainly have limited the number of participants and thus also the acceptance of the study. In addition, no project-related travel expenditures were necessary due to online implementation. The study design was developed in accordance with the Declaration of Helsinki and was submitted to the data protection officer and the institutional review board at the Medical Faculty of the University of Leipzig. The data protection conformity has been verified. After consulting our institutional review board an ethical vote was not necessary according to the Professional Code for Physicians in Germany. The data collection took place on a voluntary and anonymous basis. Consent to participate was given in each case as part of the online survey.

### Conducting Delphi rounds for EPA amelioration and consensus

The study time for one Delphi round was set at two weeks. After seven days, one electronic reminder was sent. The Delphi rounds were restricted to the following dates: 1st round 9th – 23rd September 2019, 2nd round 30th September – 14th October 2019, 3rd round 21st October -4th November 2019. The revision time by the author group did not last longer than a week.

In the first round, there was the opportunity to propose new EPA titles. These were evaluated in the following round with regard to relevance. In addition, sociodemographic data and the participants' teaching experience were requested.

Each survey was introduced by a short introductory page with explanations of the process and expected survey time. Education experts then received the descriptions of the EPAs in the current version. Relevance and the categorized content of every EPA was assessed based on a 4-point Likert scale (does not apply, does rather not apply, rather applies, applies). In addition, the participants were able to provide qualitative feedback and specific suggestions for improvement for each of the categories in an additional text box. Data collection and statistical processing was conducted with EvaSys® (version 7.1, evasys GmbH, Lüneburg, Germany) and Microsoft Excel (version 2010, Microsoft Corporation, Redmond, Washington, US).

**Fig. 1:**
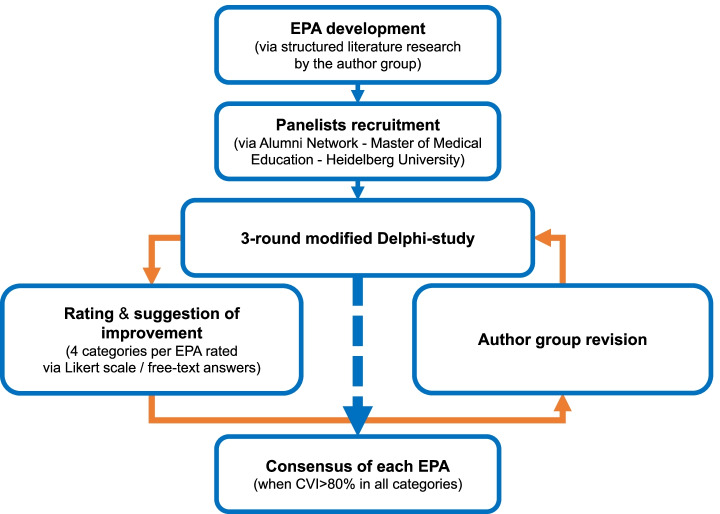
Study design flowchart /Delphi consensus procedure (CVI: context validity index)

Consent was achieved with a Content Validity Index (CVI) of ≥ 80% [37]. The CVI is the percentage of participants who agree to the content of a certain category of an EPA (rather applies, applies). If a CVI ≥ 80% was achieved for all categories of an EPA, it was considered to be consented. In this case the EPA was not evaluated further. However, each EPA went through at least one Delphi round (Fig. 1).

## Results

### Sociodemographic data

We quantitatively and qualitatively analysed 28 data sets (round 1: *n* = 12, round 2: *n* = 6, round 3: *n* = 10). Sociodemographic data are shown in Table 1.

**Table 1:** Socio-demographic data of participants

	**n**	**%**
**Gender**		
male	23	85
female	4	15
divers	0	0
**Age**		
< 30	0	0
30–40	10	36
40–50	12	43
> 50	6	21
**Clinical experience in years**		
< 5	1	4
5–10	11	39
10–15	4	14
> 15	12	43
**Active years in teaching and education**		
< 5	0	0
5–10	11	39
10–15	7	25
> 15	10	36
**Position**		
Trainee doctor	1	4
Speciality doctor	6	21
Consultant	18	64
Chief doctor or professor	0	0
Non-clinical employee	3	11
**Expertise in EPAs**		
yes	23	82
no	5	18

### Consented EPAs

During the study, the education experts developed a consensus on all 5 EPAs elaborated by the author group. Table 2 shows the final titles and their relevance with the CVI and the corresponding mean value.

**Table 2:** Elaborated EPA titles

		**Relevance of EPA**		
EPA	**Title**	Round 1 CVI (mean)	Round 2 CVI (mean)	Round 3 CVI (mean)
1	Induction of general anaesthesia in a fasting adult ASA-1 / ASA-2 patient for a low-risk procedure	100 (4,0)	100 (4,0)	/
2	Performing a preoperative evaluation in an adult ASA-1 / ASA-2 patient for a low to medium-risk procedure	90 (3,7)	100 (4,0)	/
3	Acute pain management in an adult ASA-1 / ASA-2 patient	82 (3,5)	83 (3,5)	/
4	Examination, assessment and presentation / handover of an adult patient in the intensive care unit	70 (3,0)	100 (3,8)	/
5	Initial evaluation and therapy of an acutely critically ill adult patient in a (simulated) emergency situation	91 (3,8)	100 (3,8)	100 (4,0)

Presentation of the elaborated EPAs with title as well as relevance ratings in the form of the content validity index in percent and the numerical mean. The education experts rated on a Likert scale from 1 (does not apply) to 4 (applies)

During the study, education experts not only assessed the relevance of the EPAs, but also gave quantitative and qualitative feedback on the completeness of titles, specifications and limitations, as well as knowledge, abilities, skills and professional attitudes. In addition, we evaluated qualitative comments from the participants on each EPA as a whole. By this means, the other categories of an EPA were processed. Quantitative data of EPA categories regarding titles, specifications and limitations as well as knowledge, abilities, skills and professional attitudes are shown in Table 3. After the second round, 4 of the 5 EPAs were completed with a CVI of 100% in all categories. After the third round, all EPAs in each category were successfully approved and consented.

**Table 3:** All EPAs

	**Completeness of title**			**Completeness of specifications & limitations**			**Completeness of knowledge, skills and professional attitudes**		
EPA	Round 1 CVI (mean)	Round 2 CVI (mean)	Round 3 CVI (mean)	Round 1 CVI (mean)	Round 2 CVI (mean)	Round 3 CVI (mean)	Round 1 CVI (mean)	Round 2 CVI (mean)	Round 3 CVI (mean)
1	100 (3,8)	100 (4,0)	/	82 (3,4)	100 (3,8)	/	90 (3,4)	100 (3,7)	/
2	80 (3,5)	100 (3,8)	/	91 (3,2)	100 (3,7)	/	100 (3,5)	100 (3,7)	/
3	80 (3,4)	100 (4,0)	/	73 (2,9)	100 (3,8)	/	100 (3,4)	100 (3,7)	/
4	89 (3,1)	100 (3,8)	/	40 (1,9)	100 (3,7)	/	50 (2,5)	100 (3,4)	/
5	70 (3,1)	67 (3,2)	100 (3,9)	90 (3,3)	100 (3,7)	100 (3,6)	88 (3,5)	100 (3,5)	100 (3,6)
6	/	/	100 (3,9)						
7	/	/	100 (3,9)						
8	/	/	100 (3,7)						
9	/	/	40 (2,5)						
10	/	/	80 (3,2)						
11	/	/	60 (3,1)						

Presentation of all EPAs with the round-specific evaluations in the form of the content validity index in percent and the numerical mean value for the categories title, specifications & limitations as well as knowledge, skills and professional attitudes. The education experts rated on a Likert scale from 1 (does not apply) to 4 (applies)

EPA 1 “Induction of general anaesthesia in a fasting adult ASA-1 / ASA-2 patient for a low-risk procedure” is shown as an example in Table 4. All five EPAs are listed in full in the supplement of this article.

**Table 4:** EPA 1

Title	**Induction of general anaesthesia in a fasting adult ASA-1 / ASA-2 patient for a low-risk procedure**
	**Anaesthesiology**
Specifications & limitations	1. Identification of patient, operation, side if applicable and documents using a standardized checklist including clinical evaluation of the patient to verify the preoperative patient status 2. Establishment / interpretation of basic monitoring (RR, SpO2, ECG) and intravenous access 3.Brief device check according to the recommendations of the German Society for Anaesthesiology and Intensive Care Medicine 4. Team briefing (induction and emergency strategy) 5.Induction of general anaesthesia induction with dose-appropriate use of hypnotics, opioids, muscle relaxants and perioperative antibiotic prophylaxis 6. Basic airway management a. Preoxygenation including checking FiO_2_ und etCO_2_ b. After onset of hypnosis, adequate bag mask ventilation, if applicable with aid techniques (2-hand method, oropharyngeal airway aids) c. Airway management with endotracheal tube, laryngeal mask including tube and position evaluation d. Performing a volume or pressure-controlled ventilation therapy *Context*: Elective anaesthesia induction in the operating room *Limitation*: Surgical indication urgent/emergency, age < 18 years, expected difficult airway, ≥ ASA 3, non-fasting
Competency domain	Medical Expert, Communicator, Collaborator, Professional
Knowledge, skills and professional attitudes	**Knowledge** • Anatomy of the cardiovascular system, airway and thorax • Cardiovascular und respiratory physiology • Indication, contraindication, adverse drug reactions, pharmacokinetics/-dynamics and dosage of hypnotics, opioids and muscle relaxants in anaesthesia • Predictors for assessing the depth of hypnosis / quantitative state of consciousness (e.g. RASS) • Instructions for adequate bag mask ventilation • Indication and contraindication of airway aids (Guedel/Wendl tube, laryngeal mask, endotracheal tube) • Signs of correct positioning of the endotracheal tube • Signs of adequate ventilation and placement in the case of supraglottic airway aids (e.g. bubble, suprasternal notch and performance test) • Clinical standards (Standard Operating Procedures) **Skills** • Adequate placement of a peripheral venous cannula, taking into account the cannula size and puncture site • Opening the patient’s airway (e.g. jaw thrust handle) and performing adequate bag mask ventilation, if necessary with the use of oropharyngeal airway aids • Airway management with tube, laryngeal mask or laryngeal tube • Documentation (e.g. in a patient data management system) • Performing a volume and pressure-controlled ventilation therapy **Professional attitudes** • Professional, respectful interaction and targeted communication in a team and with patients, including consideration of diversity (age, gender, origin) • Closed-loop communication technique • Comply with national hygiene guidelines • Recognition of one's own limits with regard to knowledge, abilities and skills. Continuous reflection on one's own actions and immediate request for help if necessary
Assessment	Structured oral examination, case-based discussions Observation: Mini-CEX, DOPS
Entrustment at the end of undergraduate training	**2b**

### Additionally proposed EPAs

The participants also proposed six further EPAs in the first Delphi round. In the second and third round, EPA title descriptions were evaluated and rated with regard to their relevance. The final titles are shown in Table 5 with the round-specific CVI and the corresponding mean value. A consensus was achieved for four out of six of these EPAs.

**Table 5:** Proposed EPA titles

		**Relevance of EPA**		
EPA	**Title**	Round 1 CVI (mean)	Round 2 CVI (mean)	Round 3 CVI (mean)
6	Maintenance of general anaesthesia in an adult ASA-1 / ASA-2 patient during a low to medium-risk procedure	/	100 (3,7)	100 (3,7)
7	Elimination of general anaesthesia in an adult ASA-1 / ASA-2 patient after an uncomplicated, low to medium-risk procedure	/	100 (4,0)	90 (3,5)
8	Postoperative anaesthesiologic care of an adult ASA-1 / ASA-2 patient in the post anaesthesia care unit after an uncomplicated low to medium-risk procedure	/	50 (2,8)	100 (3,6)
9	End-of-Life Decision Making	/	83 (2,7)	60 (2,6)
10	Initial sepsis management in an adult, critically ill patient	/	83 (3,0)	90 (3,6)
11	(Postoperative) transfer of an adult, ventilated patient to the intensive care bed	/	50 (2,2)	50 (2,6)

Presentation of the proposed EPAs in round 1 with titles and the round-specific relevance ratings in form of the content validity index in percent and the numerical mean. The education experts rated on a Likert scale from 1 (does not apply) to 4 (applies)

Based on qualitative feedback, the authors group had specified the titles of the proposed EPAs so that they were already consented at the end of the study. In further work, these will be elaborated in the remaining categories and fed into a Delphi process. This should be considered in future studies.

## Discussion

In recent years, EPAs have become more and more important in medical education. For example, the new national competence-based catalogue of learning objectives for medicine in Germany published in 2021 contains a total of six core EPAs that must be achieved across all disciplines at the end of medical school [38]. Our study now shows the elaboration and consent between education experts of five relevant EPAs in anaesthesiology for final-year medical students in Germany. EPA descriptions were defined and consented in an iterative, online-based process between education experts and the author group. They represent activities descriptions that should be mastered by final-year medical students under direct supervision at the end of their training in anaesthesiology. The results can easily be transferred to other medical faculties. At the same time, the EPAs also fit in well with the attempts being made in many places to implement EPAs broadly in existing medical curricula, starting with individual workplace-based assessments with nested EPAs in individual disciplines, leading to core EPAs for the entire medical curriculum.

Among the participants in the further development process of the EPAs, a low sociodemographic proportion of women of 15% was remarkable. The reasons for this are not entirely clear but are most likely due to the continuing gender inequality in the academic environment [39]. However, this should not have had an impact on the processing of the EPAs, since focus was laid primarily on didactic experiences. These can be considered sufficiently high for all participants due to the completed master's degree in medical education and an activity in undergraduate teaching of at least five years.

The greatest hurdle on the way to a consensus was the determination of the level of entrustment for each activity especially considering the legal liability after medical complications. According to current medical licensing regulations in Germany, licensed doctors are responsible for the activities of final-year medical students. Teaching doctors are currently largely on their own, without any legal and professional framework. Risk management per se and the avoidance of complications is undoubtedly a complex process in the healthcare sector: external control mechanisms such as audits or accreditations are rather perceived as disruptive methods to control risks, leaving trust is an alternative [40]. EPAs are the attempt to raise entrustable decision-making with the help of workplace-based assessments to an objective level [5]. Complications in anaesthesiology are generally rare, but they can have life-threatening consequences without adequate emergency care management. During the modified Delphi process, education experts and an author team expressed identical opinions on this matter, so that a consensus in the categories of assessment and entrustment level was found quickly. This coincides with current work from Germany: Holzhausen and colleagues published 12 approved, interdisciplinary, non-invasive EPAs for final-year medical students, which should be mastered at supervision level 3 ("indirect supervision") by the time they start working [41].

It quickly became clear that EPA complexity and limitations for final-year students had to be adjusted to a realistic level. As a result, the categories “titles” and “limitations” have been further modified and refined (Table 6). Current research shows that after the introduction of EPAs, there is often a large gap between the expectations of the curriculum developers and medical students [34]. One way to address this would be to involve students in the development process of future EPAs [42].

**Table 6:** Further development of EPA titles

EPA	Starting title	Final title
1	Induction of general anaesthesia	Induction of general anaesthesia in a fasting adult ASA-1 / ASA-2 patient for a low-risk procedure
2	Performing a preoperative evaluation	Performing a preoperative evaluation in an adult ASA-1 / ASA-2 patient for a low to medium-risk procedure
3	Acute pain management	Acute pain management in an adult ASA-1 / ASA-2 patient
4	Care of a critically ill patient	Examination, assessment and presentation / handover of an adult patient in the intensive care unit
5	Advanced Life Support in a simulated setting	Initial evaluation and therapy of an acutely critically ill adult patient in a (simulated) emergency situation

The feasibility and acceptance of the described EPAs will depend on various factors. Ten Cate and colleagues have recently pointed out that in current clinical practice, entrustment decision-making is often made ad-hoc and for a specific situation [5]. These implicit processes are only made explicit and transparent by an EPA-based curriculum and can promote acceptance [25, 43]. The continuous assessment of performance using workplace-based assessments will be a novelty for most sites, as will the assumption of responsibility taken by students. The prerequisite for the successful implementation will be an effective feedback culture and the availability of structured feedback competence [21, 31]. This must be supported and developed through fundamental integration into faculty development. The supportive learning culture describes a necessary teaching and learning environment that systematically provides the development of a trustful relationship, the promotion of an open feedback culture and fixed timeframes for feedback [44]. For this feedback, the repeated realisation of the assessments is of fundamental importance. Here, practice will show whether this can be implemented to a sufficient degree for all students for all five EPAs. In case of doubt, we believe that the number of EPAs should be reduced to be able to evaluate the remaining ones adequately and repeatedly. In order to achieve more flexibility, a broadening of assessment formats to e.g., pure knowledge-based tests or entrustment-based discussions would also be an option in some cases [45].

The described EPAs may positively influence the medical education development process and represent a valuable contribution from the perspective of medical education experts. They can act as a link between undergraduate and postgraduate training and thus prove to be a positive influence for new training regulations in the field of anaesthesiology. For this, however, it will be essential to further intensify the cooperation between medical faculties and the medical associations. The restructuring of undergraduate training of students in the final year in anaesthesiology to an EPA-based curriculum is a novelty at the University of Leipzig. Even though not all students complete a rotation in anaesthesiology in their final year of medical school and thus these are only optional specialty-specific nested EPAs, this is an important step for EPA implementation at our institution. With this work, the basis for an EPA-based curriculum was initiated. The CRU + (Curriculum Utrecht—Patient-oriented Longitudinal Utrecht Study Program) is the first to describe an EPA-based curriculum for medical students [27]. In analogy, our EPAs could represent a starting point for the development of a complete EPA-based curriculum in order to systematically merge nested EPAs into higher-level EPAs. Educationally and politically driven, competency-based medical education and EPAs will be part of the new national competency-based catalogue of learning objectives and most likely be implemented in licensing regulations for doctors in Germany by 2025/26 [46]. For this reason, a few months ago, the first core EPAs were already published by the German National Institute for State Examinations in Medicine, Pharmacy and Psychotherapy (IMPP) within the scope of the assessment framework [47]. Our nested EPAs can be a good opportunity to familiarise students and examiners with the format and to gain experience. By publishing these EPAs, these maybe considered appropriate for use in other institutions nationally and internationally. At the same time, the experience gained can also be reported back nationally, and thus meaningfully support the introduction of core EPAs within the framework of the new medical licensing regulations in the coming years.

To remain resource-efficient, this study had to be conducted in a solely online-based manner. Therefore, any misunderstandings that might have arisen during the study could not be clarified directly. However, none of the study participants made use of the opportunity to contact the study directors by phone or email, so that uncertainties were unlikely. Due to the incomplete response rate, there may have been a selection bias, as not all education experts from the alumni network participated in the study. For this reason, the generalizability and transferability to other locations with different workplace practices is only possible to a limited extent. Study participants comprised mainly speciality doctors in senior physician positions (*n* = 18, 64%) and only to a minimal extent doctors in training (*n* = 1, 4%). Students did not take part in the study. Both groups, students as well as doctors in training, would have been an important source although the majority probably had no experience in the field of EPAs. As a result, these groups could not be used for content validation. Despite these limitations, the Delphi process can be considered as a success in our opinion and the five consented EPAs can now be evaluated in student teaching. The fact that such a Delphi process is a method of choice for EPA development has meanwhile been proven by other similar studies [48, 49].

Apart from being implemented in our local undergraduate medical curriculum, our EPAs provide a basis for answering further research questions. These could include the scientific evaluation of a multi-centre implementation process or validation of assessment tools. It remains to be clarified how many EPAs can actually be taught and examined in a certain time frame without overloading the curriculum. Whether an EPA-based curriculum leads to amelioration in doctor training or better patient outcomes remains to be answered. However, the possibility of operationalized competency-based training and a broad implementation of EPAs makes it, increasingly easier to come closer to answering this question.

## Conclusion

We provide full descriptions of five relevant EPAs for final-year medical students in anaesthesiology. Development and consensus took place via a modified online Delphi process. Participants comprised medical education experts in anaesthesiology in Germany. Our EPAs represent a further step and starting point for EPA-based curricula in medical training in Germany linking undergraduate, postgraduate and specialty training.

## Supplementary Information

**Additional file 1.** 

## Data Availability

All data generated or analysed during this study are included in this published article and its supplementary information files.

## Abbreviations

AAMC: Association of American Medical Colleges
ALS: Advanced Life Support
AMEE: Association for Medical Education in Europe
ARDS: Acute Respiratory Distress Syndrome
ASA: American Society of Anaesthesiology
CAM-ICU: Confusion Assessment Method for Intensive Care Unit
CbD: Case based Discussions
CBME: Competency based Medical Education
CVVH: Continuous veno-venous hemofiltration
CVVHD: Continuous veno-venous hemodialysis
CVVHDF: Continuous veno-venous hemodiafiltration
CVI: Context Validity Index
CRM: Crew Resource Management
DOPS: Direct Observation of Procedural Skills
DGAI: Deutsche Gesellschaft für Anästhesiologie und Intensivmedizin (engl. German Society for Anaesthesiology and Intensive Care Medicine)
ECG: Electrocardiogram
ECLS/ECMO: Extracorporeal Life Support System/Extracorporeal Membrane Oxygenation
EPA: Entrustable Professional Activities
ERC: European Resuscitation Council
FAST: Focused Assessment with Sonography for Trauma
FATE: Focused assessed trans-thoracic Echocardiography
IMPP: German National Institute for State Examinations in Medicine, Pharmacy and Psychotherapy
ISBAR: Acronym: Identification, Situation, Background, Assessment, Recommendation
KDiGO: Kidney Disease: Improving Global Outcomes
LEMON: Acronym: Look, Evaluate, Mallampati, Obstruction, Neck mobility
Mini-CEX: Mini Clinical Evaluation Exercise
MME: Master of Medical Education
PONV: Postoperative Nausea and Vomiting
RASS: Richmond Agitation Sedation Scale
SAMPLER: Acronym: Symptoms, Allergies, Medication, Past medical history, Last meal, Events prior to incident, Risk factors
SOPs: Standard Operation Procedures
WHO: World Health Organization
