# Supplementary material for: Development and consensus of entrustable professional activities for final-year medical students in anaesthesiology
Source: BMC Anesthesiol. 2022 Apr 29;22:128. doi: 10.1186/s12871-022-01668-8 (PMC9052481; doi:10.1186/s12871-022-01668-8)
Supplement: Supplementary file 1 — Additional file 1. [file 12871_2022_1668_MOESM1_ESM.pdf]

Supplement

EPA 1

Title	**Induction of general anaesthesia in a fasting adult ASA-1 / ASA-2 patient for a low-risk procedure**
	**Anaesthesiology**
Specifications & limitations	1. Identification of patient, operation, side if applicable and documents using a standardized checklist including clinical evaluation of the patient to verify the preoperative patient status<br>2. Establishment / interpretation of basic monitoring (RR, SpO2, ECG) and intravenous access<br>3. Brief device check according to the recommendations of the German Society for Anaesthesiology and Intensive Care Medicine<br>4. Team briefing (induction and emergency strategy)<br>5. Induction of general anaesthesia induction with dose-appropriate use of hypnotics, opioids, muscle relaxants and perioperative antibiotic prophylaxis<br>6. Basic airway management<br>a. Preoxygenation including checking FiO_2 und $etCO_2$<br>b. After onset of hypnosis, adequate bag mask ventilation, if applicable with aid techniques (2-hand method, oropharyngeal airway aids)<br>c. Airway management with endotracheal tube, laryngeal mask including tube and position evaluation<br>d. Performing a volume or pressure-controlled ventilation therapy<br><br>*Context*: Elective anaesthesia induction in the operating room<br>*Limitation*: Surgical indication urgent/emergency, age <18 years, expected difficult airway, ≥ASA 3, non-fasting
Competency domain	Medical Expert, Communicator, Collaborator, Professional
Knowledge, skills and professional attitudes	**Knowledge**<br>• Anatomy of the cardiovascular system, airway and thorax<br>• Cardiovascular und respiratory physiology<br>• Indication, contraindication, adverse drug reactions, pharmacokinetics/-dynamics and dosage of hypnotics, opioids and muscle relaxants in anaesthesia<br>• Predictors for assessing the depth of hypnosis / quantitative state of consciousness (e.g. RASS)<br>• Instructions for adequate bag mask ventilation<br>• Indication and contraindication of airway aids (Guedel/Wendl tube, laryngeal mask, endotracheal tube)<br>• Signs of correct positioning of the endotracheal tube<br>• Signs of adequate ventilation and placement in the case of supraglottic airway aids (e.g. bubble, suprasternal notch and performance test)<br>• Clinical standards (Standard Operating Procedures)<br><br>**Skills**<br>• Adequate placement of a peripheral venous cannula, taking into account the cannula size and puncture site<br>• Opening the patient’s airway (e.g. jaw thrust handle) and performing adequate bag mask ventilation, if necessary with the use of oropharyngeal airway aids<br>• Airway management with tube, laryngeal mask or laryngeal tube<br>• Documentation (e.g. in a patient data management system)<br>• Performing a volume and pressure-controlled ventilation therapy<br><br>**Professional attitudes**<br>• Professional, respectful interaction and targeted communication in a team and with patients, including consideration of diversity (age, gender, origin)<br>• Closed-loop communication technique<br>• Comply with national hygiene guidelines<br>• Recognition of one's own limits with regard to knowledge, abilities and skills. Continuous reflection on one's own actions and immediate request for help if necessary
Assessment	Structured oral examination, case-based discussions<br>Observation: Mini-CEX, DOPS
Entrustment at the end of undergraduate training	**2b**

EPA 2

Title	**Performing a preoperative evaluation in an adult ASA-1 / ASA-2 patient for a low to medium-risk procedure**
	**Anaesthesiology**
Specifications & limitations	1. Taking an anaesthetic history and performing a physical examination, incl.<br>a. Evaluation of difficult airways predictors (e.g. LEMON)<br>b. Aspiration risk evaluation<br>c. Perioperative medication management (anticoagulation, antidiabetic drugs, cardiovascular concomitant medication, premedication)<br>d. Evaluation of PONV risk according to the APFEL score<br>e. Taking a structured coagulation history incl. laboratory interpretation and action plan (e.g. anaemia diagnostics) if applicable<br>f. ASA risk classification<br>2. Development of a surgical and patient-adapted anaesthesia concept<br>3. Documentation (e.g. in a patient data management system and information sheet)<br>4. Conducting an anaesthesiologic informed consent discussion with the patient<br><br>*Context*: Anaesthesia outpatient department<br>*Limitation*: ≥ASA 3, surgical indication urgent/emergency, age <16 years, anxious and legal cared for patients
Competency domain	Medical Expert, Communicator, Collaborator, Manager, Health Advocate, Professional
Knowledge, skills and professional attitudes	**Knowledge**<br>• Airway anatomy<br>• Pathophysiology of anaesthesiologic relevant diseases (e.g. arterial hypertension, diabetes mellitus, coronary heart disease, ...)<br>• Normal values of vital parameters and laboratory chemistry<br>• Predictors for difficult airway, risk of aspiration, PONV risk, and ASA classification<br>• Perioperative medication management and premedication<br>• Anaesthesia procedure and complications including frequency of occurrence<br>• Patient fasting criteria<br>• Clinical standards (Standard Operating Procedures)<br><br>**Skills**<br>• Performing an anaesthesiologic anamnesis<br>• Conducting an anaesthesiologic informed consent education incl. relevant complication using a structured information sheet<br>• Patient-adapted, professional and structured interviewing technique<br>• Clinical examination relevant to anaesthesia (incl. auscultation of the lungs / heart and examination of the dental status)<br>• Documentation (e.g. in a patient data management system and information sheet)<br><br>**Professional attitudes**<br>• Professional, respectful interaction and targeted communication in a team and with patients, including consideration of diversity (age, gender, origin)<br>• Comply with national hygiene guidelines<br>• Recognition of one's own limits with regard to knowledge, abilities and skills. Continuous reflection on one's own actions and immediate request for help if necessary
Assessment	Structured oral examination, case-based discussions<br>Observation: Mini-CEX, DOPS
Entrustment at the end of undergraduate training	**2b**

EPA 3

Title	**Acute pain management in an adult ASA-1 / ASA-2 patient**
	**Pain Medicine**
Specifications & limitations	1. Taking a structured pain history<br>2. Development of a pain therapy concept taking into account the type of pain and previous pain history of the patient:<br><br>*Context*: Patient care in the post anaesthesia care unit and acute pain service<br>*Limitation*: Chronic pain patients, patients with pain medication and drug addiction, age <18 years
Competency domain	Medical Expert, Communicator, Collaborator, Manager, Health Advocate, Professional
Knowledge, skills and professional attitudes	**Knowledge**<br>• Forms of pain (pain quality, quantity, localization, head's zones)<br>• Pain scales<br>• Differential diagnosis of pain<br>• Local, systemic (including patient-controlled analgesia) and regional pain therapy (regional anaesthesia)<br>• WHO pain level scheme and opioid equivalence<br>• Indication, contraindication, adverse drug reactions, pharmacokinetics/dynamics and dosage of local anaesthetics, non-opioid, low- and high-potency opioid analgesics as well as co-analgesics and adjuvants in pain medicine<br>• Pain pump and pain catheter therapy incl. peridural anaesthesia techniques<br>• Clinical standards (Standard Operating Procedures)<br><br>**Skills**<br>• Taking a structured pain history<br>• Management of a patient-controlled pain pump and pain catheters incl. peridural anaesthesia techniques<br>• Documentation (e.g. in a patient data management system)<br><br>**Professional attitudes**<br>• Professional, respectful interaction and targeted communication in a team and with patients, including consideration of diversity (age, gender, origin)<br>• Comply with national hygiene guidelines<br>• Recognition of one's own limits with regard to knowledge, abilities and skills. Continuous reflection on one's own actions and immediate request for help if necessary
Assessment	Structured oral examination, case-based discussions<br>Observation: Mini-CEX, DOPS
Entrustment at the end of undergraduate training	**2b**

EPA 4

Title	**Examination, assessment and presentation / handover of an adult patient in the intensive care unit**
	**Intensive Care Medicine**
Specifications & limitations	1. Performing an organ-oriented, focused clinical examination on the (unconscious) patient<br>2. Structured, organ-oriented status documentation taking into account vital and ventilation parameters, laboratory chemistry and ongoing therapies (e.g. catecholamine therapy, dialysis)<br>3. Structured patient presentation on ward rounds and handover at shift change using the ISBAR concept<br>4. Working in an interdisciplinary and interprofessional team in the intensive care environment<br><br>*Context*: Daily routine care of critically ill patients<br>*Limitation*: age <18 years, patients in severe shock, patients on ECLS/ECMO therapy, patients with irreversible brain dysfunction
Competency domain	Medical Expert, Communicator, Collaborator, Manager, Scholar, Professional
Knowledge, skills and professional attitudes	**Knowledge**<br>• Clinical presentation, typical findings, classifications and therapeutic principles of common diseases and complications in the intensive care unit<br>○ Qualitative and quantitative states of consciousness including delirium (GCS, RASS classification, CAM-ICU) and cerebral pathologies<br>○ Kidney failure (KDIGO guideline), liver failure, lung failure and ARDS (Berlin criteria), shock (hypovolaemic, cardiogenic, distributive, obstructive)<br>○ Sepsis and multiple organ failure<br>○ Polytrauma<br>• Pathogenetic principles and pathomechanisms as a basis for understanding and differential diagnosis of life-threatening diseases<br>• Physiology of the critically ill patient<br>• Principles of analgesic sedation including indication, contraindication, adverse drug reactions, pharmacokinetics/-dynamics and dosage of typical hypnotics and analgesics<br>• Principles of organ replacement therapy<br>○ Principles of ventilation therapy in lung-healthy and ARDS patients<br>○ Principles of shock and catecholamine therapy<br>○ Principles of renal replacement therapy (CVVH, CVVHD, CVVHDF and intermittent procedures)<br>• Nutritional management: principles of enteral and parenteral nutrition<br>• Structural processes on the ward (e.g. curve guidance, admission and discharge management), clinical standards (Standard Operating Procedures) and quality criteria for the intensive care unit<br>• National guidelines if applicable<br><br>**Skills**<br>• Structured physical examination of an (unconscious) patient<br>• Interpretation of vital (monitoring) and ventilation parameters, as well as laboratory chemistry and radiological findings (sonography, X-ray, CT/MRT)<br>• Performing a patient presentation and handover according to the ISBAR concept<br>• Management of medical devices for infusions, suction drains (e.g. thorax), central venous access, arterial catheters, urinary bladder catheters, gastric tubes<br>• Intra- (e.g. handover, consultation) and interprofessional communication techniques (e.g. treatment plan communication with nursing staff, physiotherapists and social workers) as well as patient-adapted communication strategies<br>• Documentation (e.g. in a patient data management system) and setting daily goals<br><br>**Professional attitudes**<br>• Professional, respectful interaction and targeted communication in a team and with patients, including consideration of diversity (age, gender, origin)<br>• Comply with national hygiene guidelines<br>• Recognition of one's own limits with regard to knowledge, abilities and skills. Continuous reflection on one's own actions and immediate request for help if necessary
Assessment	Structured oral examination, case-based discussions<br>Observation: Mini-CEX, DOPS
Entrustment at the end of undergraduate training	**2b**

EPA 5

<table>
<tr><td>Title</td><td>Initial evaluation and therapy of an acutely critically ill adult patient in an (simulated) emergency situation</td></tr>
<tr><td></td><td>Emergency Medicine / Anaesthesiology</td></tr>
<tr><td>Specifications & limitations</td><td>1. Recognizing an emergency situation with typical clinical presentation
a. Cardiac arrest
b. Chest pain (e.g. acute coronary syndrome)
c. Tachycardiac and bradycardiac arrhythmias (e.g. ventricular tachycardia, atrial fibrillation, AV block)
d. Anaphylaxis
e. Shortness of breath and hypoxemia disorders
f. Disorders of metabolism and electrolyte imbalance (especially hypo- and hyperglycaemia and hypo- and hyperkalaemia)
g. Acute neurological deficit and seizure
2. Performing of an initial clinical and apparatus evaluation incl. developing an emergency action plan
3. Early request for assistance

Context: Emergency team training in a simulated environment
Limitation: Age <16 years, psychiatric emergencies</td></tr>
<tr><td>Competency domain</td><td>Medical Expert, Communicator, Collaborator, Manager, Professional</td></tr>
<tr><td>Knowledge, skills and professional attitudes</td><td>Knowledge
• Clinical presentation, examination results and therapy principles of typical emergency situations as well as differential diagnoses of key emergency medical symptoms
o Cardiopulmonary resuscitation algorithm according to current guidelines (e.g. ERC guidelines)
o Treatment principles and differential diagnoses of acute chest pain, acute dyspnoea, acute neurological deficit and generalized seizures
o Treatment principle for malignant cardiac arrhythmias (e.g. ERC algorithm for periarrest arrhythmias)
o Treatment principle for anaphylaxis (e.g. ERC algorithm)
o Kidney failure (KDIGO guideline) and hyperkalaemia management
• Indication, contraindication, adverse drug reactions, pharmacokinetics/-dynamics and dosage of typical emergency drugs (incl. basic principles of emergency anaesthesia)
• Basics of CRM and knowledge of "human factors"
• Clinical standards (Standard Operating Procedures)

Skills
• Establishing and evaluating standard monitoring, if applicable with a defibrillator and an i.v./i.o. access
• Extension of monitoring and evaluation based on differential diagnosis:
o Recording and interpretation of a 12-lead ECG
o Assessment of a venous and arterial blood gas analysis incl. blood sugar
• Hygienically and dose-appropriate application of i.v./i.o. emergency medication
• Application modes of O_2, bag-mask ventilation and basic airway management as well as their optimization
• Structured handover of the emergency situation using a standardized scheme (e.g. ISBAR)
• a. Detection of cardiac arrest and performing a high-quality cardiopulmonary resuscitation according to current guidelines (e.g. ERC)
• b.-g. Taking a focused anamnesis using to a standardized scheme (e.g. SAMPLER), clinical and technical examination using the ABCDE algorithm as well as the situation and guideline-based conduction of an initial emergency therapy in a typical non cardiac arrest emergency situation

Professional attitudes
• Professional, respectful interaction and targeted communication in a team and with patients, including consideration of diversity (age, gender, origin)
• Comply with national hygiene guidelines
• Recognition of one's own limits with regard to knowledge, abilities and skills. Continuous reflection on one's own actions and immediate request for help if necessary
• Consideration of the CRM principles</td></tr>
<tr><td>Assessment</td><td>Structured oral examination, case-based discussions
Observation with video analysis and structured feedback (debriefing), Mini-CEX, DOPS</td></tr>
<tr><td>Entrustment at the end of undergraduate training</td><td>2b</td></tr>
</table>
